# Constitutive interferon signaling maintains critical threshold of MLKL expression to license necroptosis

**DOI:** 10.1038/s41418-018-0122-7

**Published:** 2018-05-21

**Authors:** Joseph Sarhan, Beiyun C. Liu, Hayley I. Muendlein, Chi G. Weindel, Irina Smirnova, Amy Y. Tang, Vladimir Ilyukha, Maxim Sorokin, Anton Buzdin, Katherine A. Fitzgerald, Alexander Poltorak

**Affiliations:** 1 Medical Scientist Training Program (MSTP), Tufts University School of Medicine, Boston, MA 02111 USA; 2 Graduate Program in Immunology, Tufts University Sackler School of Biomedical Sciences, Boston, MA 02111 USA; 3 Graduate Program in Genetics, Tufts University Sackler School of Biomedical Sciences, Boston, MA 02111 USA; 4 Department of Microbial Pathogenesis and Immunology, Texas A&M Health Science Center, Bryan, TX 77808 USA; 5 Department of Immunology, Tufts University School of Medicine, Boston, MA 02111 USA; 6 Petrozavodsk State University, Petrozavodsk, Republic of Karelia 185910 Russia; 7 National Research Center, Kurchatov Institute, Moscow, Russian Federation; 8 I.M. Sechenov First Moscow State Medical University, Moscow, Russian Federation; 9 Program in Innate Immunity, University of Massachusetts Medical School, Worcester, MA 01605 USA; 10 Centre for Molecular Inflammation Research, Department of Cancer Research and Molecular Medicine, NTNU, 7491 Trondheim, Norway

**Keywords:** Cell death and immune response, Interferons

## Abstract

Interferons (IFNs) are critical determinants in immune-competence and autoimmunity, and are endogenously regulated by a low-level constitutive feedback loop. However, little is known about the functions and origins of constitutive IFN. Recently, lipopolysaccharide (LPS)-induced IFN was implicated as a driver of necroptosis, a necrotic form of cell death downstream of receptor-interacting protein (RIP) kinase activation and executed by mixed lineage kinase like-domain (MLKL) protein. We found that the pre-established IFN status of the cell, instead of LPS-induced IFN, is critical for the early initiation of necroptosis in macrophages. This pre-established IFN signature stems from cytosolic DNA sensing via cGAS/STING, and maintains the expression of MLKL and one or more unknown effectors above a critical threshold to allow for MLKL oligomerization and cell death. Finally, we found that elevated IFN-signaling in systemic lupus erythematosus (SLE) augments necroptosis, providing a link between pathological IFN and tissue damage during autoimmunity.

## Introduction

Necroptosis is a form of necrotic cell death that occurs downstream of receptor-interacting protein kinases (RIP1/RIP3) activation and disruption of the plasma membrane by the pseudokinase mixed lineage kinase like-domain (MLKL) [1–3]. If virus infection inhibits apoptosis, necroptosis becomes a secondary mode of cell death to limit viral replication [4, 5]. In the laboratory, the use of the pan-caspase inhibitor zVAD with TNFα stimulation drives RIP3/MLKL dependent necroptosis [4, 6]. Recently, necroptosis was shown to occur downstream of toll-like receptor (TLR) stimulation with zVAD. TLR3/4 are unique in inducing necroptosis via a common endosomal adaptor, TRIF (TIR-domain-containing adapter-inducing interferon-β) [6, 7] independently of TNFα. Upon engagement of TLR3/4, TRIF interacts with RIP1/RIP3 via RHIM domains, an event critical for necroptosis [7–9]. Notably, activation of TLR3/4 also induces interferon (IFN) production through TRIF [10]. With the finding that type I IFN receptor deficient cells are resistant to TLR3/4 mediated necroptosis, TRIF was attributed the additional role of mediating necroptosis via IFN induction [11, 12].

Type I interferons (IFN-I) comprise a family of 14 α isoforms and one β, κ, ω, and δ isoform each, all of which bind a common heterodimeric receptor, IFN-αR1 and IFN-αR2 (IFNAR) [13]. IFNs regulate host immune responses by binding the receptor and activating STAT1/2 transcription factors to regulate a diverse family of genes termed interferon stimulated genes (ISGs) [14]. IFN-I is induced downstream of cytosolic nucleic acid sensing or endosomal TLRs. In contrast, constitutive IFN-I signaling in the resting state is less characterized, despite having been detected in human and mouse cells including MEFs [15], B cells, and macrophages [14]. It has been suggested that constitutive IFN sustains the expression of JAKs, STATs, and other IFN-I signaling components. Another function of constitutive IFN-I is to maintain basal levels of immune-related genes to enable a rapid response to viral infection [16].

Sufficient IFN signaling is critical for host defense to avoid lethal viral and mycobacterial infections such as in STAT1 deficient individuals [17]. In contrast, excessive IFN has been linked to pathology in multiple autoimmune diseases including lupus, psoriasis, Sjogrens, dermatomyositis, and type I diabetes [18]. In the case of interferonopathies including Aicardi–Goutières syndrome and STING-associated vasculopathy with onset in infancy (SAVI), IFN directly drives disease [19]. Recently, necroptosis has been linked to both IFN signaling and autoimmune disease [20, 21]. Although IFNAR-deficient cells are resistant to TLR-induced necroptosis, the mechanism underlying this protection as well as any connection of these findings to autoimmunity remains unclear.

In this study, we found that constitutive IFN signaling is crucial for the initiation of necroptosis via the endogenous activity of the cytosolic DNA sensing pathway cGAS/STING. Our findings prompt the model that constitutive IFN signaling drives the steady-state expression of MLKL to enable a rapid necroptosis response.

## Results

### Constitutive IFN signaling licenses necroptosis in macrophages

**Fig. 1:**
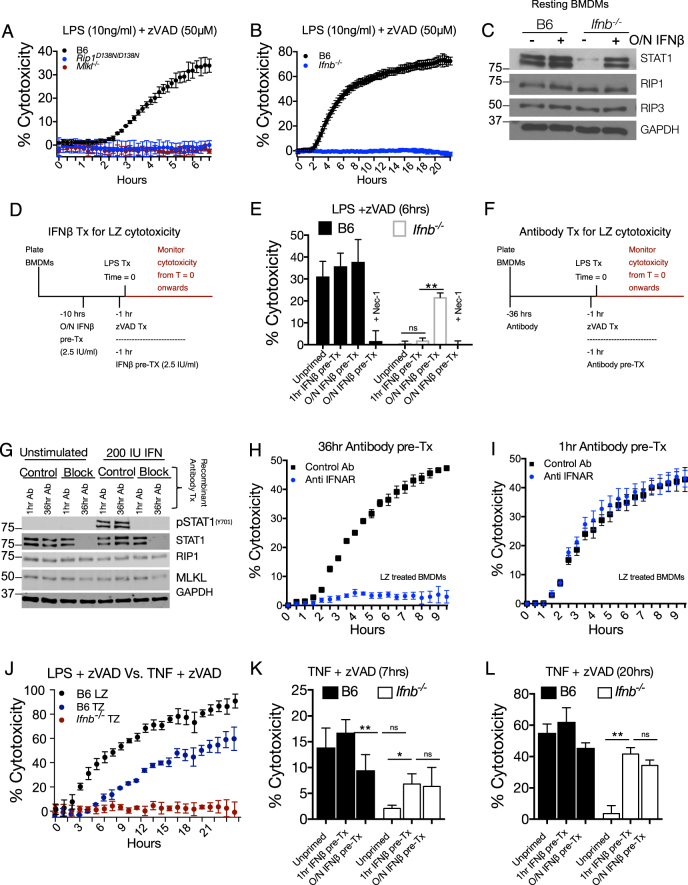
Constitutive type I interferon (IFN-I) signaling is required for initiation of necroptosis. **a**, **b** Propidium iodide incorporation as a readout of cytotoxicity, measured every 15 min following LPS/zVAD treatment of BMDMs of indicated genotype. **c** Western blot of indicated proteins of resting BMDMs from B6 and *Ifnb*^*−/−*^ with or without overnight interferon treatment (2.5 IU IFNβ/ml). **d** Treatment scheme for overnight or 1 h treatment of BMDMs with 2.5 IU/ml of recombinant IFNβ. **e** Viability of B6 and *Ifnb*^*−/−*^ BMDMs measured 6 h after LPS /zVAD treatment. **f** Treatment scheme for overnight or 1 h antibody treatment of B6 BMDMs. **g** Western blot for indicated proteins from B6 BMDMs treated with antibodies as in (**f**) before stimulation with 200 IU/ml IFNβ for 30 min. **h**, **i** Propidium iodide incorporation of B6 BMDMs treated with LZ and antibody pre-treatment for 36 h (**h**) or 1 h (**i**) as in (**f**). **j** Propidium iodide incorporation over 22 h of B6 BMDMs treated with LPS/zVAD or TNF (50 ng/ml)/zVAD (50 μM) and *Ifnb*^*−/−*^ BMDMs treated with TNF/zVAD. **k**, **l** Cytotoxicity measurement at 7 h (**k**) or 20 h (**l**) from B6 and *Ifnb*^*−/−*^ BMDMs treated with TNF/zVAD with IFN pre-treatment as in (**d**). All LPS/zVAD treatments were: LPS (10 ng/ml) and zVAD (50 μM/ml). In all cases of IFNβ pre-treatment (overnight or 1 h), the IFNβ is washed away before addition of experimental conditions. Time point cytotoxicity quantification ± SD from three independent experiments compared using student two tailed *t*-test: ns non-significant (*p* > 0.05); *p < 0.05; **p < 0.01; ***p < 0.001. All western blot and kinetic cytotoxicity data are representative of three or more experiments. See related supplementary Figure 1

To measure cytotoxicity, we monitored the kinetics of propidium iodide uptake in real time, with LPS and zVAD (LZ) treatment (Fig. 1a). Compared to wild-type cells, BMDMs from either kinase inactive RIPK1 *Rip1*^*D138N/D138N*^, *Mlkl*^*−/−*^ (Fig. 1a) or RIP3 RHIM domain mutant (*Ripk3*^*ΔR/ΔR*^) mice (Figure S1A) were resistant to LPS/ zVAD cytotoxicity.

The role of IFN in necroptosis has been studied using IFNAR-deficient animals which are unresponsive to both inducible (stimulation dependent) and constitutive (resting state) IFN [22]. To distinguish between inducible and constitutive IFN, we used *Ifnb*^*−/−*^ BMDMs, which were completely resistant to LZ-induced necroptosis (Fig. 1b). In the resting state, *Ifnb*^*−/−*^ BMDMs had lower ISG expression, including STAT1 and STAT2 (Fig. 1c, Figure S1B), and *Isg15*, *Irf7* and *Mx1* mRNA (Figure S1C). This ISG deficiency can be rescued by overnight treatment with a low dose (2.5 IU/ml) of recombinant IFNβ (Fig. 1c, Figure S1B, C). The reduction of ISG expression at both the mRNA and protein level is consistent with low-constitutive IFN in *Ifnb*^*−/−*^ BMDMs. Unlike STAT1, levels of RIP1, RIP3, and IRF3 proteins were not substantially affected by constitutive IFN (Fig. 1c, Figure S1B). In agreement with recently published work [22], we observed a reduction of MLKL levels in IFN-deficient BMDMs. This reduction in MLKL expression in *Ifnb*^*−/−*^ BMDMs was rescued with low dose (2.5 IU/ml) of recombinant IFNβ treatment overnight (Figure S1D).

To study the role of constitutive IFN on cell viability, we used overnight (10 h) or 1-h pre-treatment of cells with low dose (2.5 IU/ml) IFNβ prior to stimulation with LZ (Fig. 1d). At 6 h of LZ stimulation, only overnight IFNβ treatment sensitized *Ifnb*^*−/−*^ BMDMs to necroptosis (Fig. 1e). We observed rapid upregulation of ISGs with IFN treatment (Figure S1E), suggesting that time is required for ISGs to be translated. We also used IFNAR blocking antibody to inhibit either stimulus-induced IFN (−1 h pre-treatment) or constitutive IFN (−36 h Antibody) (Fig. 1f). Both 1 h and 36-h treatment of blocking antibody inhibited STAT1 phosphorylation in B6 BMDMs when stimulated with IFNβ (Fig. 1g). However, only 36-h pre-treatment decreased total STAT1 (Fig. 1g). Correspondingly, 36-h blockade of IFNAR abolished necroptosis in B6 BMDMs (Fig. 1h), whereas 1-h blockade did not affect necroptosis (Fig. 1i).

Extending our findings to other models of necroptosis, we treated BMDMs with TNF/zVAD (TZ) and observed that cytotoxicity is slower than with LZ necroptosis (Fig. 1j). As with LZ-induced necroptosis, *Ifnb*^*−/−*^ BMDMs were resistant to TZ-induced death (Fig. 1j). Interestingly, unlike LZ-induced necroptosis, TZ-necroptosis was sensitized by either overnight or 1 h IFNβ pre-treatment *Ifnb*^*−/−*^ BMDMs (Fig. 1k, l). Correspondingly, 24 h of IFNAR blockade protected against TZ-induced cell death, whereas 1 h did not (Figure S1F). We also investigated the SMAC-mimetic (SM) and zVAD model of necroptosis, which unlike TNF and zVAD, resulted in the same kinetics and magnitude of cell death as LPS and zVAD treatment (Figure S1G). Like LZ treatment, *Ifnb*^*−/−*^ BMDMs were resistant to SM + zVAD-induced cytotoxicity and could be sensitized to necroptosis after overnight treatment with low dose IFNβ (Figure S1H).

Extending our findings to human monocyte derived macrophages (MDM), we confirmed that LPS and zVAD induces RIPK-dependent cell death using Necrostatin-1 (Figure S1I). Next, we blocked IFN signaling with the JAK1/2 inhibitor Ruxolitinib (Rux) in MDMs either 1-h or 36 h prior to LZ stimulation. Chronic Rux treatment downregulated *IRF7* expression in resting cells (Figure S1J). Furthermore, 36 h but not 1 h Rux pre-treatment downregulated total STAT1 levels, although both treatments effectively inhibited STAT1 phosphorylation in response to exogenous IFNβ (Figure S1K). Rux treatment did not affect MLKL levels in MDMs (Figure S1K). Consistent with mouse macrophages, chronic Jak inhibition protected MDMs against LZ-induced necroptosis (Figure S1L, M). Treatment of MDMs with IFNAR blocking antibody partially inhibited STAT1 phosphorylation in response to exogenous IFNβ (Figure S1N). However, 36-h treatment with IFNAR blocking antibody did not reduce total STAT1 levels (Figure S1N) or IRF7 expression (Figure S1O). Accordingly, 36 h IFNAR blocking antibody did not protect human MDMs from LZ treatment (Figure S1P).

### The endosomal TLR4 adaptor TRIF initiates necroptosis independently of IFNβ induction

**Fig. 2:**
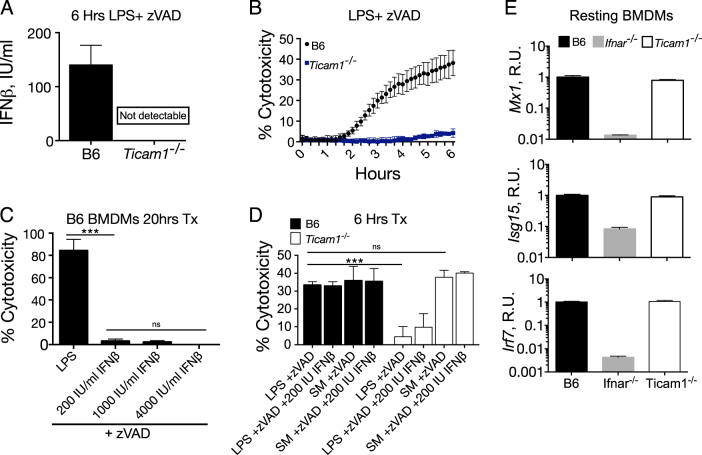
The endosomal TLR4 adaptor TRIF initiates necroptosis independently of LPS-induced IFNβ. **a** Supernatant IFNβ cytokine measured by ELISA from B6 and *Ticam1*^*−/−*^ BMDMs stimulated with LPS/zVAD for 6 h. **b** Propidium iodide incorporation following LPS/zVAD treatment of B6 and *Ticam1*^*−/−*^ BMDMs as a readout of cytotoxicity. **c** Cytotoxicity by PI incorporation of B6 BMDMs treated for 20 h with LPS/zVAD or varying doses of IFNβ and zVAD. **d** Propidium iodide incorporation of B6 and *Ticam1*^*−/−*^ BMDMs 6 h following LPS/zVAD or SM (1 nM)/zVAD treatment with or without 200 IU IFNβ. **e** Quantitative PCR of *Irf7*, *Isg15*, and *Mx1* mRNA relative to *Gapdh* from resting B6, *Ifnar*^*−/−*^ and *Ticam1*^*−/−*^ BMDMs. All LPS/zVAD treatments were: LPS (10 ng/ml) and zVAD (50 μM/ml). Time point cytotoxicity quantification ± SD from three independent experiments compared using student two tailed *t*-test: ns is non-significant (*p* > 0.05); *p < 0.05; **p < 0.01; ***p < 0.001. All qPCR and kinetic cytotoxicity data are representative of three or more experiments. See related supplementary Figure 2

TRIF (encoded by*Ticam1*), a key TLR4 adaptor, is required for both LPS-induced IFN (Fig. 2a) and LPS/zVAD driven cell death (Fig. 2b). During LZ-induced necroptosis, TRIF is known to interact with RIP1/RIP3 via RHIM domain interactions [7]. The resistance of *Ticam1*^*−/−*^ BMDMs to cell death was attributed to their inability to produce IFN upon LPS stimulation [11], leading to a model that necroptosis can be driven by TRIF-mediated IFN. In B6 BMDMs, we found that IFNβ, even when used at supraphysiologic doses of up to 4000 U/ml, is not sufficient to drive necroptosis in the context of caspase inhibition (Fig. 2c). To determine whether TRIF dependent IFN production is necessary for LPS-induced necroptosis, we treated B6 and *Ticam*^*−/−*^ BMDMs with high-dose exogenous IFNβ (200 U/ml) concurrent with LZ stimulation (Fig. 2d) and found no sensitization of cell death. Furthermore, we found that TRIF deficient cells were fully sensitive to TLR-independent, SM induced necroptosis (Fig. 2d). *Ticam1*^*−/−*^ BMDMs also exhibited normal levels of ISGs at steady state (Fig. 2e).

The discrepancy between our data and published reports regarding the necroptotic potential of (IFN + zVAD) treatment may be explained by endotoxin contamination of BSA, a carrier frequently used to dilute recombinant IFN. We re-constituted IFN in 0.1% BSA from either endotoxin free BSA or Fisher brand BSA to a concentration of 10 IU IFNβ/ml and measured cell death (Figure S2A) and TNF production (Figure S2B) with IFN/zVAD treatment, both of which were un-detectable with endotoxin free BSA.

### The DNA sensing pathway cGAS/STING is required for constitutive interferon production

**Fig. 3:**
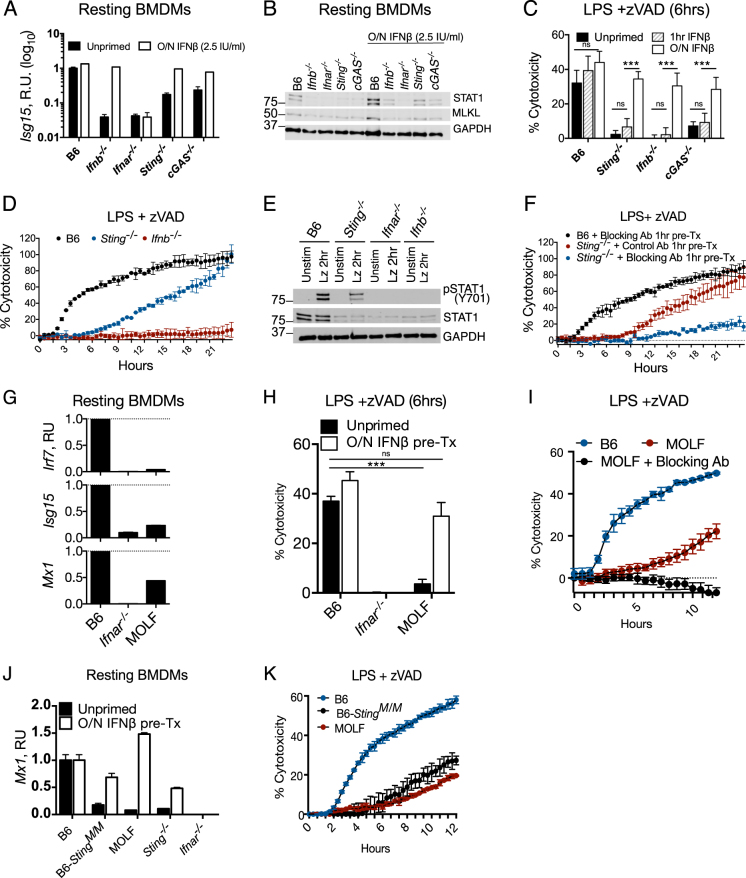
Cytosolic DNA sensing via cGAS and STING is required for constitutive interferon production. **a** Quantitative PCR of *Isg15* and western blot of indicated proteins (**b**) from resting B6, *Ifnb*^*−/−*^, *Ifnar*^*−/−*^, *Sting*^*−/−*^, and *cGAS*^*−/−*^ BMDMs that were untreated or pre-treated with IFNβ overnight (2.5 IU IFNβ/ml). **c** Propidium iodide incorporation 6 h following LPS /zVAD treatment of BMDMs, either not treated with IFN (unprimed), pre-treated with 2.5 IU IFNβ/ml overnight or 1 h before LPS treatment. **d** Twenty-two hours time course of propidium iodide incorporation of B6, *Sting*^*−/−*^, *Ifnb*^*−/−*^, BMDMs stimulated with LPS/zVAD. **e** Western blots of pSTAT1 and STAT1 from BMDMs of indicated genotype either unstimulated or 2 h LPS/zVAD stimulated. **f** B6 and *Sting*^*−/−*^ BMDMs pre-treated 1 h with MAR1 IFNAR blocking or isotype control antibody and monitored for LPS/zVAD-induced cytotoxicity. **g** Quantitative PCR of ISG mRNA relative to GAPDH mRNA from resting B6, *Ifnar*^*−/−*^ and MOLF BMDMs. **h** Propidium iodide incorporation 6 h following LPS /zVAD treatment of B6, *Ifnar*^*−/−*^ and MOLF BMDMs with or without overnight interferon pre-treatment (2.5 IU IFNβ/ml). **i** Propidium iodide incorporation during 12 h of stimulation with LZ of B6 and MOLF BMDMs with or without 1 h MAR1 IFNAR blocking antibody pre-treatment. **j** Quantitative PCR of MX1 mRNA relative to GAPDH mRNA from resting BMDMs of indicated genotype without overnight 2.5 IU IFNβ/ml. **k** Propidium iodide measurement over 12 h of LPS/zVAD treatment of B6, N10 congenic B6 *Sting*^*MOLF/MOLF*^, and MOLF BMDMs. All LPS/zVAD treatments were: LPS (10 ng/ml) and zVAD (50 μM/ml). In all cases of IFNβ pre-treatment (overnight or 1 h), the IFNβ is washed away before addition of experimental conditions. Time point cytotoxicity quantification ± SD from three independent experiments compared using student two tailed *t*-test: ns is non-significant (*p* > 0.05); *p < 0.05; **p < 0.01; ***p < 0.001. All western blot and kinetic cytotoxicity data are representative of three or more experiments. See related supplementary Figure 3

Aberrant DNA presence, either from the nucleus [23], mitochondria [24], or lysosomes [25], can activate cytosolic DNA sensing via cGAS and STING to upregulate IFN. Indeed, BMDMs lacking STING and the DNA sensor cGAS had reduced levels of *Isg15*, *Irf7*, and *Mx1* mRNAs and low levels of STAT1 protein (Fig. 3a, b; Figure S3A, B). Consistent with the defect in ISG signature, STING- and cGAS-deficient BMDMs exhibited resistance to LZ-induced early-onset (6 h) necroptosis (Fig. 3c), which was not due to a deficiency in RIP1, RIP3, or Ticam1 expression (Figure S3C).

Interestingly, untreated *Sting*^*−/−*^ BMDMs became sensitized to cytotoxicity over time, in contrast to the complete resistance of *Ifnb*^*−/−*^ BMDMs (Fig. 3d). We observed STAT1 phosphorylation in response to LZ treatment in *Sting*^*−/−*^ BMDMs, despite low STAT1 (Fig. 3e). *Sting*^*−/−*^ and *cGAS*^*−/−*^ BMDMs were next stimulated with LZ in the presence of IFNAR blocking antibody MAR1 or isotype control antibody (antibodies were added 1 h prior to LZ stimulation). Blocking IFNAR during LZ stimulation protected both *Sting*^*−/−*^ and *cGAS*^*−/−*^ BMDMs from necroptosis, but did not affect cell death in B6 BMDMs (Fig. 3f; Figure S3D). Additionally, we found that *Sting*^*−/−*^ and *cGAS*^*−/−*^ BMDMs were completely resistant to TNF/zVAD-induced cell death (Figure S3E, F).

Extending our findings to TLR3 mediated necroptosis with polyinosinic-polycytidylic acid (pI:C) and zVAD, *Ifnar*^*−/−*^ BMDMs were resistant to cell death, and *Sting*^*−/−*^ and *cGAS*^*−/−*^ BMDMs were kinetically delayed (Figure S3G).

We additionally found that IFNβ production is dampened in *Sting*^*−/−*^ BMDMs compared to B6 macrophages in response to both LPS and LZ (Figure S3H, I), but this effect of constitutive IFN did not apply to TNFα (Figure S3J, K).

### Low IFN-signaling status confers resistance to necroptosis in MOLF/Ei macrophages

We previously reported that resistance to necroptosis in peritoneal macrophages of wild-derived MOLF mice are conferred by downregulation of the deubiquitinase CYLD [26]. Like peritoneal macrophages, MOLF BMDMs were also resistant to LZ-induced necroptosis (Figure S3L), however uniform CYLD expression (Figure S3M) suggested another mechanism for resistance to necroptosis. Despite low levels of ISGs, (Fig. 3g), MOLF macrophages readily underwent STAT1 phosphorylation in response to exogenous IFNβ1 (Figure S3N). Like other constitutive IFN deficient cells, MOLF BMDMs exhibited slow necroptosis kinetics, resembling *Sting*^*−/−*^ and *cGAS*^*−/−*^ BMDMs (Fig. 3f), and can be sensitized to necroptosis with overnight IFN priming (Fig. 3h). Similarly, IFNAR blockade during LZ stimulation prevented late time point sensitization to necroptosis in MOLF macrophages (Fig. 3i).

To examine the contribution of the hypomorphic MOLF allele of *Sting* [27], we tested BMDMs from mice that are congenic for the MOLF allele of *Sting* on the C57BL/6 background (B6-*Sting*^*M/M*^*)* for cell death and IFN status. Indeed, we observed low-constitutive IFN in B6-*Sting*^*M/M*^ BMDMs (Fig. 3j; Figure S3O) and protection from necroptosis (Fig. 3k).

### Interferon receptor signaling is required for RIP1 and RIP3 degradation and MLKL S345 phosphorylation in BMDMs

**Fig. 4:**
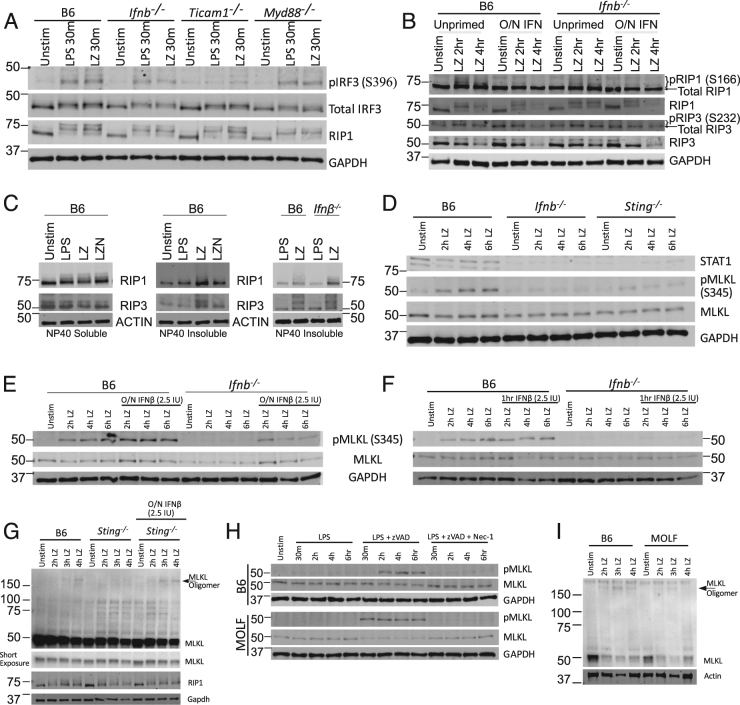
Interferon receptor signaling is required for MLKL phosphorylation in B6 BMDMs a western blots of pIRF3 (S396), total IRF3, RIP1, and GAPDH from unstimulated, 30 min LPS or LPS/zVAD stimulated BMDMs from B6, *Ifnb*^*−/−*^, *Ticam1*^*−/−*^ and *Myd88*^*−/−*^ mice. **b** Western blots of indicated proteins from B6 and *Ifnb*^*−/−*^ BMDMs with or without overnight 2.5 IU/ml IFNβ treated with LPS/zVAD. **c** Western blots of RIP1 and RIP3 kinases in BMDMs of indicated genotypes in NP40 soluble or insoluble fractions. **d** Western blots of indicated proteins from B6, *Ifnb*^*−/−*^, and *Sting*^*−/−*^ BMDMs treated with LPS/zVAD for indicated duration of time. **e**–**f** Western blots of phospho MLKL (pMLKL), MLKL and GAPDH from B6 and *Infb*^*−/−*^ BMDMs that were untreated or primed with 2.5 IU IFNβ/ml overnight. (**e**) or 1 h (**f**) and treated with LPS/zVAD. **g** Non-reducing western blot featuring MLKL oligomers from B6 and *Sting*^*−/−*^ BMDMs (with or without 2.5 IU/ml IFNβ overnight) treated with LPS /zVAD for indicated times. **h** B6 and MOLF BMDMs were treated with LPS, LPS/zVAD or LPS/zVAD/Nec-1 for indicated durations and analyzed for indicated proteins by western blotting. Nec-1 was used at a concentration of 30 μM. **i** Non-reducing western blot featuring MLKL oligomers from B6 and MOLF BMDMs treated with LPS /zVAD for indicated times. All LPS/zVAD treatments were: LPS (10 ng/ml) and zVAD (50 μM/ml). In all cases of IFNβ pre-treatment (overnight or 1 h), the IFNβ is washed away before addition of experimental conditions. All western blot data are representative of three or more independent experiments. See related supplementary Figure 4

To determine the step at which constitutive IFN is needed for LZ necroptosis, we looked at the initiating events in LPS signaling, including TLR4 endocytosis followed by engagement of TRIF and IRF3 phosphorylation [28]. Here we observed that TRIF dependent Serine 396 IRF3 phosphorylation following LPS/zVAD stimulation was similar in all strains including *Ifnb*^*−/−*^ BMDMs (Fig. 4a; Figure S4A). Additionally, the RIP1 mobility shift suggestive of phosphorylation [11] was also present in *Ifnb*^*−/−*^ (Fig. 4a) and dependent on a kinase recruited to either TRIF or MyD88 (Figure S4B).

To investigate the activity of RIP1 and RIP3, we established which LZ-induced modifications of RIP1 and RIP3 are reversible by the RIP1 inhibitor Nec-1. We found that RIP1 Serine 166 phosphorylation, as well as depletion of RIP1 and RIP3 total protein, were reversed by Nec-1 (Figure S4C). We could not detect LPS/zVAD-induced RIP3 modifications including the activating phospho serine 232 [29] in B6 BMDMs. We found that *Ifnb*^*−/−*^ BMDMs undergo RIP1 S166 phosphorylation and overnight IFN treatment, attenuates this phosphorylation (Fig. 4b). Unlike B6 BMDMs, *Ifnb*^*−/−*^ BMDMs do not downregulate RIP1 and RIP3 unless primed overnight with IFN (Fig. 4b). Next we utilized NP-40 detergent insolublecellular lysates to look for RIP1 and RIP3 modifications in amyloid-like “necrosomes” [30]. Consistent with published reports [31], RIP modifications were present in the NP-40 necrosomes in a RIP kinase dependent manner (Fig. 4c; S4D). RIP1 and RIP3 modifications in the necrosomes were not reduced in *Ifnb*^*−/−*^ BMDMs (Fig. 4c).

Moving onto MLKL modification, consistent with published work [22] we found that MLKL Serine 345 phosphorylation is deficient in *Ifnb*^*−/−*^ and *Sting*^*−/−*^ BMDMs (Fig. 4d). The loss-of-MLKL phosphorylation in *Ifnb*^*−/−*^ BMDMs was rescued with overnight but not acute low-dose IFNβ treatment (Fig. 4e, f). Furthermore, we found that 36 h—but not 1 h—treatment with IFNAR blocking antibody reduced MLKL phosphorylation (Figure S4F,G). Downstream of phosphorylation, MLKL forms oligomers, detectable by non-reducing SDS-PAGE (Figure S4H). Consistent with defective MLKL phosphorylation, *Sting*^*−/−*^*, Ifnar*^*−/−*^, and *cGAS*^*−/−*^ BMDMs were deficient in oligomerization (Fig. 4g; Figure S4I,J).

On the C57BL/6 genetic background, constitutive IFN is required upstream of MLKL phosphorylation. In contrast to this, MOLF BMDMs, robustly phosphorylated MLKL, even in the absence of constitutive IFN (Fig. 4h; Figure S4K). Interestingly, MOLF BMDMs only weakly oligomerize MLKL (Fig. 4i).

### Critical threshold of MLKL expression determines oligomerization

**Fig. 5:**
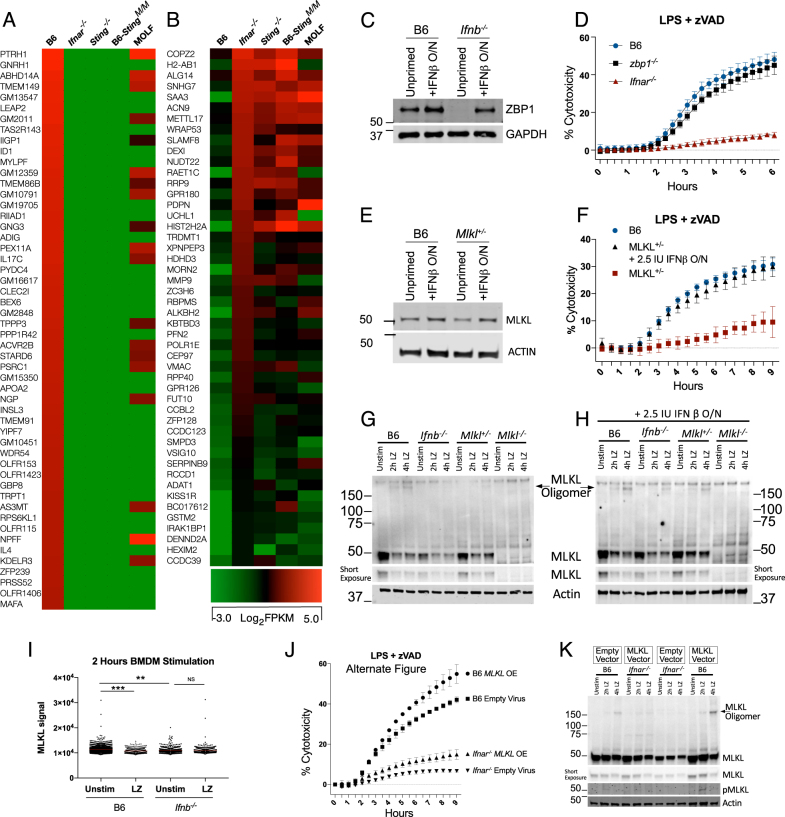
Critical threshold of MLKL expression determines oligomerization potential. **a** RNA sequencing of resting BMDMs showing genes downregulated in *Ifnar*^*−/−*^, *Sting*^*−/−*^, *Sting*^*MOLF/MOLF*^, and MOLF compared to B6 (**b**) and significantly higher in expression compared to B6. **c** Western blot of ZBP1 in non-treated or O/N 2.5 IU/ml IFNβ treated B6 and *Ifnb*^*−/−*^ BMDMs. **d** Six hours time course of viability of LPS/zVAD treated B6, *Zbp1*^*−/−*^, and *Ifnar*^*−/−*^ BMDMs. **e** Western blot of MLKL in non-treated or O/N 2.5 IU/ml IFNβ treated B6 and *Mlkl*^*+/−*^ BMDMs. **f** Nine hours time course of viability of LPS/zVAD treated B6 and *MLKL*^*+/-*^ BMDMs. **g**, **h** Non-reducing western blot featuring MLKL oligomers from BMDMs of indicated genotype treated with LPS /zVAD either non-IFNβ primed (**g**) or treated overnight with 2.5 IU/ml IFNβ (**h**). **i** High-content imaging of endogenous MLKL in PFA fixed B6 and *Ifnb*^*−/−*^ BMDMs treated 2 h with LPS/zVAD. **j**, **k** B6 and Ifnar^*−/−*^ BMDMs transduced with either empty virus or MLKL over expressing virus and analyzed for LZ-induced cytotoxicity (**j**) and non-reducing western blots for MLKL oligomerization and phosphorylation (**k**). All LPS/zVAD treatments were: LPS (10 ng/ml) and zVAD (50 μM/ml). In all cases of IFNβ pre-treatment (overnight or 1 h), the IFNβ is washed away before addition of experimental conditions. MLKL signal compared using student two tailed *t*-test: ns is non-significant (*p* > 0.05); *p < 0.05; **p < 0.01; ***p < 0.001. All western blot and kinetic cytotoxicity data are representative of three or more experiments. See related supplementary Figure 5

To screen for ISGs in the necroptosis pathway, we compared resting gene expression of B6 and constitutive IFN deficient BMDMs. We identified ~80 genes that are commonly downregulated in resting *Ifnar*^*−/−*^, *Sting*^*−/−*^, and B6-*Sting*^*M/M*^ BMDMs when compared to B6 (Fig. 5a) and ~ 50 genes upregulated in the absence of constitutive IFN (Fig. 5b). Known inhibitors of necroptosis including Ppm1b [32], TRAF2 [33], CHIP [34] and components of the ESCRT machinery [35] are unaltered by constitutive IFN status (Figure S5A,B).

Sorting genes (FPKM > 4) by differential expression between B6 and *Ifnar*^*−/−*^ BMDMs, we found *Zbp1* (Figure S5C), which can interact with RIP3 to induce necroptosis during development [36, 37] or in response to influenza A virus infection [38]. We observed that ZBP1 protein levels depended on constitutive IFN signaling (Fig. 5c). However, *Zbp1*^*−/−*^ BMDMs were not protected from LPS-induced necroptosis (Fig. 5d) or MLKL phosphorylation (Figure S5D). We also checked the ISG expression status in resting *Zbp*^*−/−*^ cells since ZBP1 was initially characterized as a cytosolic DNA sensor [39], and found no defect in the basal expression of multiple ISGs in *Zbp1*^*−/−*^ BMDMs (Figure S5E).

MLKL also appears within our genome wide analysis comparing resting gene expression in constitutive IFN deficient BMDMs (Figure S5C). Additionally, we have seen lower baseline expression of MLKL in multiple instances in (Figs. 1g, 3b, 4d, e, f and Figure S1D,S4F). We found that BMDMs from MLKL hemizygous animals (*Mlkl*^*+/−*^) were defective in baseline levels of MLKL (Fig. 5e). Interestingly, *Mlkl*^*+/−*^ BMDMs were resistant to both LPS + zVAD (Fig. 5f) and SM-164 + zVAD (Figure S5F) induced necroptosis. We found that *Mlkl*^*+/−*^ BMDMs were not deficient in MLKL phosphorylation (Figure S5G), but were deficient in MLKL oligomerization (Fig. 5g), which can be rescued with low-dose IFNβ priming (Fig. 5h).

Imaging endogenous MLKL we found a gradient of expression in resting cells, which is significantly lower in *Ifnb*^*−/−*^ compared to B6 BMDMs (Fig. 5i). We observed selective loss-of-cells expressing more MLKL in B6 BMDMs after LPS + zVAD treatment (Fig. 5i; Figure S5H).

We also overexpressed MLKL in IFNAR-deficient BMDMs to see if MLKL alone is sufficient to restore cell death. There was limited re-sensitization toward cell death (Fig. 5j; Figure S5I) or oligomerization, even though resting expression of MLKL was restored in *Ifnar*^*−/−*^ BMDMs (Fig. 5k). Together these results show that adequate MLKL expression is critical but not sufficient for necroptosis without IFNAR signaling.

### Elevated steady-state IFN predispose cells to necroptosis

**Fig. 6:**
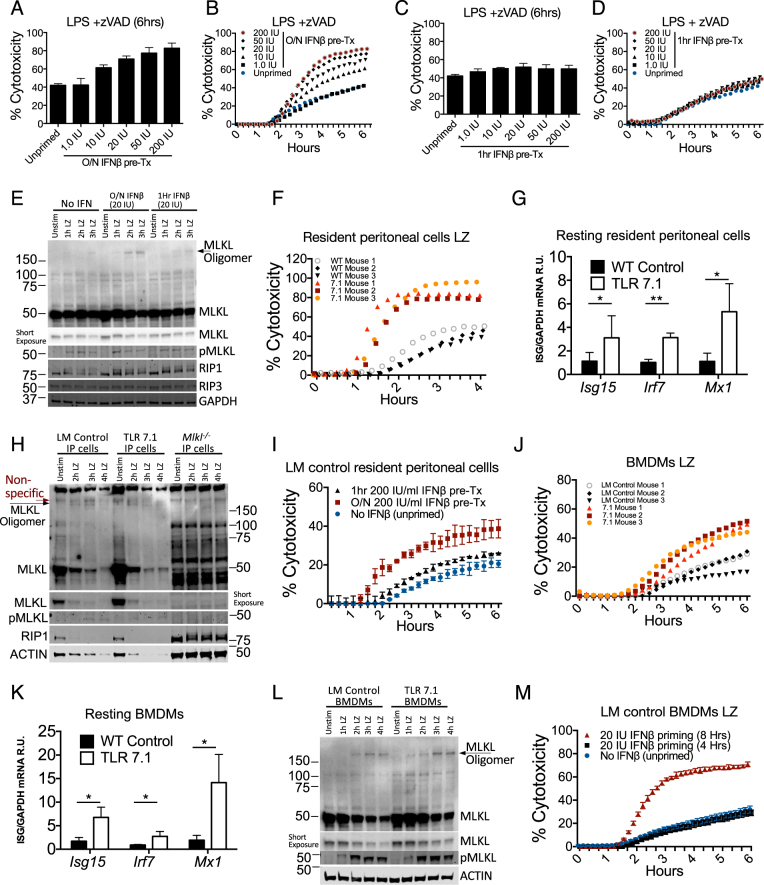
High levels of type I interferon (IFN-I) predispose cells to necroptosis. **a**, **b** Propidium iodide incorporation of B6 BMDMs at 6 h (**a**) or kinetically over 6 h (**b**) of LPS/zVAD treatment with overnight pre-treatment of recombinant IFNβ. **c**, **d** Propidium iodide incorporation of B6 BMDMs at 6 h (**c**) or kinetically over 6 h (**d**) of LPS/zVAD treatment with 1 h pre-treatment of recombinant IFNβ. **e** Western blots of multiple proteins with LPS/zVAD treatment of B6 BMDMs with indicated IFNβ pre-treatment. **f** Propidium iodide incorporation over 4 h of TLR7.1 and littermate control resident peritoneal cells treated with LPS/zVAD. **g** Quantitative PCR of ISG mRNA relative to *Gapdh* from resting TLR7.1 and littermate control resident peritoneal cells. **h** Western blots of multiple proteins with LPS/zVAD treatment of resident peritoneal cells from TLR 7.1, littermate control and Mlkl^*−/−*^ animals. **i** Propidium iodide incorporation over 6 h of TLR7.1 littermate control resident peritoneal cells either non-primed or 200 IU/ml IFNβ treated for 1 h or overnight and treated with LPS/zVAD. **j** Propidium iodide incorporation over 6 h of TLR7.1 and littermate control BMDMs treated with LPS/zVAD. **k** Quantitative PCR ISG mRNA relative to GAPDH from resting TLR7.1 and littermate control BMDMs. **l** Western blots of multiple proteins with LPS/zVAD treatment of BMDMs from TLR 7.1 or littermate control animals. **m** Propidium iodide incorporation over 6 h of TLR7.1 littermate control BMDMs either non-interferon primed or 20 IU/ml IFNβ treated for 4 or 8 h and treated with LPS/zVAD. All LPS/zVAD treatments were: LPS (10 ng/ml) and zVAD (50 μM/ml). In all cases of IFNβ pre-treatment (overnight or 1 h), the IFNβ is washed away before addition of experimental conditions. MLKL oligomers were visualized using non-reducing western blots. Cytotoxicity data are representative of three or more experiments. qPCR represent SD from three independent experiments and statistical significance was determined using Student's two tailed *t*-test: *p < 0.05; **p < 0.01; ***p < 0.001

Patients with autoimmunity have elevated circulating IFNs [40], which we predicted may increase sensitivity to necrotic stimuli. We found a dose dependent increase in LZ cytotoxicity with overnight influenza A virus infection IFNβ pre-treated B6 BMDMs (Fig. 6a, b). In contrast, exposure to these same doses of IFNβ 1 h prior to LZ did not affect cytotoxicity (Fig. 6c, d). Additionally, MLKL expression and oligomerization was enhanced during overnight of 20 IU/ml IFNβ priming (Fig. 6e), while RIP1 and RIP3 seemed unaffected. Next, we utilized a well-established model of lupus of TLR7 overexpression (TLR 7.1) [41]. Using resident peritoneal cells to more closely capture the in vivo autoimmune milieu, we found that resident peritoneal cells from TLR 7.1 animals were more sensitive to LZ-induced necroptosis and elevated ISGs relative to littermate controls (Fig. 6f, g). Additionally, we observed that MLKL expression was higher in resting TLR 7.1 resident peritoneal cells (Fig. 6h). ACTIN depletion, associated with necrotic cell death was also more pronounced in TLR 7.1 cells. We next primed TLR7.1 littermate control resident peritoneal with 200 IU IFNβ overnight, and observed an elevated necroptosis response (Fig. 6i). We also found that TLR7.1 BMDMs similarly exhibited higher necroptosis and ISGs compared to littermate controls (Fig. 6j, k). Furthermore, MLKL expression is elevated in resting TLR 7.1 BMDMs and oligomer formation is initiated faster and appears more robust (Fig. 6l). Additionally, BMDMs from TLR 7.1 littermate controls (LM) can be sensitized to undergo maximal cell death with as little as 20 IU IFNβ, for 8 h prior to LZ stimulation (Fig. 6m). Together, these results show that elevated IFN-signaling, such as in the case of autoimmunity, can sensitize cells to necroptosis.

## Discussion

**Fig. 7:**
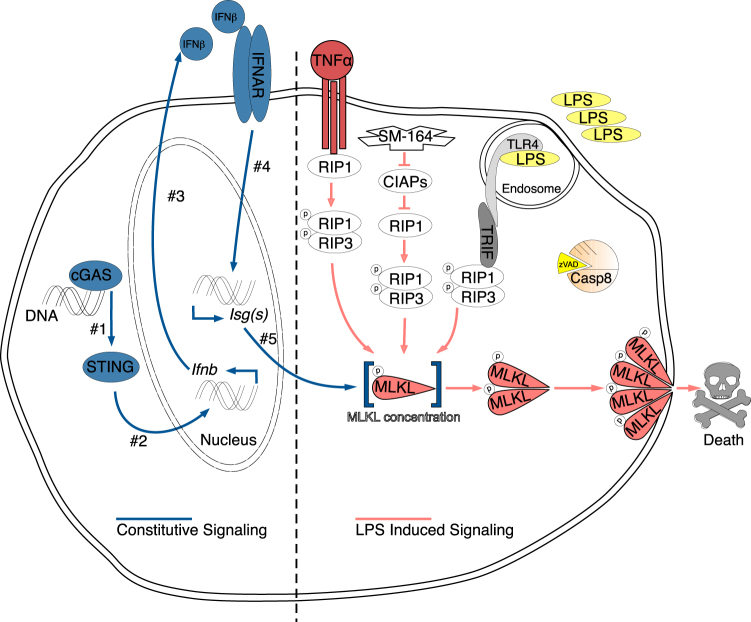
Model for the role of interferon in necroptosis Constitutive IFN signaling (left side, chronologically numbered) is driven by homeostatic cGAS/STING activation from cytosolic DNA sensing. This IFN signaling loop sustains the baseline expression of ISGs (#5). One of these ISGs is MLKL, which must be present at adequate levels to allow for MLKL oligomerization following phosphorylation. Total levels of MLKL are another critical checkpoint of regulation for necroptosis. The requirement for constitutive IFN signaling for cell death is common to LPS, SMAC-mimetic and TNF-induced necroptosis all of which also require caspase-8 inhibition as indicated by zVAD

Our findings prompt a new model of the necroptosis pathway (Fig. 7). During homeostatic cellular turnover, DNA, possibly from DNA damage-repair [23] or mitochondrial stress [24], activates the cGAS/STING pathway leading to constitutive IFN production that feeds back onto cells to sustain expression of many ISGs. MLKL is one such ISG which must be expressed sufficiently to facilitate oligomerization (Fig. 5e) and cell death (Fig. 5f). Since MLKL overexpression could not restore necroptosis in *Ifnar*^*−/−*^ macrophages (Fig. 5j), we postulate the importance of another ISG for RIPK-dependent phosphorylation of MLKL to execute necroptosis. Because RIP3 can phosphorylate MLKL, it is possible that the ISG(s) in question serve(s) a scaffolding function to bring together RIP3 and MLKL for proper necrosome formation [30]. Alternatively, constitutive IFN upregulate an inhibitor of necroptosis, though multiple known inhibitors are unaltered by constitutive IFN status (Figure S5A,B). Further investigation of differentially expressed ISGs by focusing on the intersections of B6, *Ifnar*^*−/−*^ and *Sting*^*−/−*^ will be a powerful approach to identify new regulators of necroptosis.

Previous work showed that caspase inhibition in the context of viral infection increases constitutive IFN production via the STING/cGAS pathway [24, 42, 43]. Elevated constitutive IFN may then function to increase the necroptosis potential to limit viral replication. Parallel study in the lab (Liu et al., submitted) found that constitutive IFN governs the rate of pyroptosis in response to *Legionella pneumophila* by maintaining guanylate binding protein expression. A common theme thus emerges, in which the role of IFN in both cell death pathways is in maintaining expression of critical effectors for cell death. For necroptosis, we refer to these ISGs as constitutive IFN-regulated effectors of necroptosis as CIREN(s). CIREN abundance determines the rapidity of necroptosis. Macrophages with sufficient levels of CIRENs undergo necroptosis as early as 2 h after LPS/zVAD. In contrast, lack of baseline expression of ISGs in MOLF, *cGas*^*−/−*^ and *Sting*^*−/−*^ BMDMs significantly delays onset of cell death despite ability of these BMDMs to induce IFN. This temporal defect in necroptosis can be explained by time that is required for de novo synthesis of CIREN proteins downstream of LPS-induced IFN feedback. Therefore, not only did we elucidate a role for constitutive IFN signaling in licensing necroptosis by maintenance of yet unknown but crucial players of the pathway, we also discovered that de novo IFN induction only becomes relevant in cases where constitutive IFN is lacking, to replenish the abundance of said CIREN(s). We identified MLKL as a CIREN, but we and others have shown that MLKL overexpression in IFNAR-deficient BMDMs is not sensitizing to necroptosis [22] suggesting that there are other unknown CIREN(s).

While loss-of-constitutive IFN protected cells from necroptosis, elevated IFN signaling sensitizes TLR7.1 macrophages to necroptosis, which may be relevant to elevated IFN levels in human lupus. Necrotic cell death has been proposed as a mechanism by which release of nuclear antigens and DAMPs feed the cycle of SLE pathogenesis [44]. Although, the source of necrotic cell death in lupus is unclear, we show that high-IFN levels pre-disposes macrophages to necrosis by which nuclear and cytosolic content can be released. Recent report by the Kelliher group has shown that hyper-activation of necroptosis leads to autoimmunity [21]. Here we show the inverse, in which cells from mice with autoimmunity are more sensitive to necroptosis (Fig. 6). Further work will be needed to establish the causality between necroptosis and autoimmunity.

## Materials and methods

### Reagents

Lipopolysaccharide (LPS) (*Escherichia coli* 0111:B4) was purchased from Sigma and used at 10 ng/ml. zVAD.fmk was purchased from ApexBio and used at 50 μM. Necrostatin-1 was purchased from Sigma and used at 10 μM. SM-164 was purchased from ApexBio and used at 1 μM. Poly(I:C) was from InvivoGen and used at 25 μg/ml. Recombinant mouse IFNβ (12400-1) and recombinant human IFNβ (11415-1) were purchased from PBL Assay Science and used at various concentrations as indicated. In all cases of IFNβ pre-treatment (overnight or 1 h), the IFNβ is washed away before addition of experimental conditions. Recombinant moue TNF was purchased from PeproTech and used at a concentration of 50 ng/ml. Ruxolitinib was purchased from cayman chemical and used at a concentration of 10 μM. Blocking antibody to mouse IFNAR (MAR1-5A3) and control IgG were purchased from BD Pharmingen and used at 20 μg/ml. Blocking antibody to human IFNAR (#21385–1) and control IgG were purchased from PBL and used at a concentration of 20 μg/ml. Recombinant proteins were re-constituted in 0.1% endotoxin free BSA from Akron Biotech (AK8917).

### Cell viability assays

BMDMs were plated in 96-well plates at 1 × 10^5^ cells per well in RPMI, 10% FBS, 1%PS. Cells were plated on tissue culture treated clear bottom plates (Costar 3603) in media containing 10 μg/mL propidium iodide (Life Technologies, P3566). TECAN Infinite 200 Pro plate reader was used to maintain temperature at 37 °C and 5% CO_2_ during infection. Propidium iodide uptake was measured every 15 min using bottom read using 535 nm excitation and 617 nm emission. Wells containing 0.1% Triton X-100 lysed cells were used as 100% cytotoxicity controls. Treatment + zVAD PI incorporation was relative to treatment alone (LPS + zVAD relative to LPS alone). A similar protocol was used previously [45]. For polyI:C induced necroptosis, kinetic microscopy was performed using the Cytation3 Imager (BioTek) and nuclei positive of propidium iodide signal were enumerated. Plate reading function was not used in this case, since PI intercalates with polyI:C, generating high background signal.

### Animals

C57BL/6, MOLF/Ei, Ifnar^*−/−*^ (B6.129S2-Ifnar1tm1Agt/Mmjax), Trif-/-(C57BL/6J-Ticam1Lps2/J), MyD88-/-(B6.129P2(SJL)-Myd88tm1.1Defr/J), strains were obtained from the Jackson Laboratory. Tlr7.1 Tg mice [41] (C57BL/6-Tg(Tlr7)1Boll) were obtained from Dr. B. Huber (Tufts). Ifnb^*−/−*^ were a gift from Dr. S. Vogel (UMaryland). Trif^*−/−*^/MyD88^*−/−*^, cGAS^*−/−*^ and RIP3^*−/−*^/Caspase-8^*−/−*^ animals were a gift from Dr. K. Fitzgerald (UMass). RIP3^*−/−*^/Caspase-8^*−/−*^ animals [46] were generated by Dr. D. Green (St. Jude). RIP1 kinase inactive (Rip1D138N/D138N) [47] and Mlkl^*−/−*^ animals were a gift from Dr. M. Kelliher (UMass). Mlkl^*−/−*^ [48] animals were generated by Dr. W. Alexander (Walter and Eliza Hall Institute). Zbp1^*−/−*^ mice [49] were a gift from Dr. S. Balachandran. Sting^*−/−*^ animals were a gift from Dr. G. Barber (UMiami). RIP3 RHIM domain mutant (Ripk3ΔR/ΔR) mice [50] were a gift from Dr. Francis Chan. All genetically modified mice were fully backcrossed to the C57BL/6 background. B6. MOLF-Tmem173molf (STING^*MOLF/MOLF*^) congenic mice were established by backcrossing 10 generations to C57BL/6^28^. All mice were housed in a pathogen-free facility at the Tufts University School of Medicine and experiments were performed in accordance with regulations and approval of the Tufts University Institutional Animal Care and Use Committee.

### RNA preparation and analysis

A total of 5 × 10^5^ cells were plated on 24-well TC treated plates and cells were lysed with Trizol (Invitrogen) followed by RNA extraction according to the manufacturer’s instructions. cDNA was synthesized using M-MuLV reverse transcriptase, RNase inhibitor, random primers 9, and dNTPs (New England BioLabs). cDNA was analyzed for mRNA expression levels using SYBR Green (Applied Biosystems) and intron spanning primers. Gapdh was used to normalize mRNA expression and post amplification melting curve analysis was used for all transcripts to verify specificity.

### Mouse bone marrow derived macrophages (BMDMs)

Bone marrow was isolated by flushing femurs with cold RPMI. Cell pellets were re-suspended in BMDM differentiating media (RPMI with l-glutamine, 20% FBS, 28% l-cell conditioned media, 1% Pen-Strep) and cultured for 7 days at 37 °C in 5% CO_2_ to mature to macrophages. Matured BMDMs were rested overnight in RPMI containing 10% FBS, 2% Penn/Strep, in the absence of l-cell conditioned media prior to experiments.

### Human monocyte derived macrophages (MDMs)

De-identified human peripheral blood was purchased from the New York Biologics as approved by the Institutional Review Board and Institutional Biosafety Committee at Tufts University. Monocytes were obtained from peripheral blood using the EasySep Direct Monocyte Isolation Kit from STEMCELL technologies (#19669). Monocytes were extracted and differentiated into macrophages over the course of 7 days in RPMI containing 20% FBS, 2% Penn/Strep, and 100 μg/ml of human M-CSF (PeproTech). Matured MDMs were rested for 36 h in RPMI containing 10% FBS, 2% Penn/Strep, in the absence of M-CSF prior to experiments. Similar protocols were used previously [51].

### Western blotting

To prepare whole-cell lysates, cells were lysed directly in 1× Laemmli Buffer with 5% β−mercaptoethanol, boiled for 10 min, and incubated on ice for 10 min. Protein lysates were resolved on a 10% Bis-Tris SDS gel and transferred to a nitrocellulose membrane. Blocking conditions were TBS-T with 5% BSA. Primary antibodies were diluted to 1:1000 in TBS-T with 1% BSA, and infrared (700 or 800 nm) secondary antibodies were diluted 1:30,000. Membranes were incubated with protein-specific primary antibodies overnight at 4 °C followed by 40-min room temperature incubation with secondary infrared antibodies and imaged using an Odyssey® CLx Imaging System. Image analysis was done using Image Studio software. STAT1(#9172), STAT2 (#72604) pSTAT1-Tyr701(#9167), RIP1 (#D94C12), phospho-RIP1-S166 (#65746), phospho-p38 MAPK-Thr180/Tyr182 (#9211), IRF-3 (#4302), phospho-IRF-3- Ser396 (#4947) GAPDH (#2118), and ACTIN (#3700) antibodies were purchased from Cell Signaling. RIP3 antibody (#2283) was purchased from ProSci. Total MLKL antibody was purchased from Millipore (#MABC604). Phospho-MLKL- S345 (#ab196436), and phospho-RIP3- S227 (#ab209384) antibodies were purchased from Abcam. CYLD antibody (clone 733) was purchased from Invitrogen. ZBP1 antibody (clone Zippy-1) was purchased from Adipogen. For visualizing MLKL oligomers, samples were processed in the same way as for a standard western blot but Laemmli Buffer was free from β−mercaptoethanol. MLKL was detected using the same antibody from Millipore (#MABC604), and anti-rat HRP (CST #7077) 1:2000 dilution. HRP was developed using SuperSignal™ West Femto (34095) from Thermo and visualized using a BioRAD ChemiDoc digital chemiluminescence imager.

### High-content imaging

Cells were seeded at a density of 0.5 × 10^6^ cells/cm^2^ on MatriPlate 0.17 mm glass bottom plates (DOT Scientific) in RPMI containing 10% FBS. At indicated time points post treatment, cells were fixed for 10 min with 4% paraformaldehyde, then washed with blocking buffer (10% goat serum, 2% FBS in PBS) 3× followed by 1 h room temperature incubation with blocking buffer. Overnight 4 °C incubation with anti MLKL antibody from Millipore (#MABC604) (1:250 dilution) followed by 3× blocking buffer wash and 1 h incubation with goat anti-rat AF488 Thermo (#A-11006) (1:250 dilution). Nuclei were counter-stained with Hoechst [2 μM final] Thermo (#62249) for 10 min at room temperature flowed by 3× BPS wash. For washing procedure, imaging plate was first centrifuged at 200×*g* for 5 min, then plate was dumped to remove liquid instead of aspiration to avoid removing loosely adhered cells. A total of 25 fields at ×20 magnification were captured and stitched by the Cytation3 automated microscope, generating fields of view with ~3000 cells each for image quantification. Signal intensity for MLKL was analyzed with the Gen5 software using MLKL^*−/−*^ macrophages as negative controls to determine the non-specific background signal intensity.

### MLKL overexpression

The open reading frame of murine MLKL was cloned into pLEX lentiviral plasmid using BamHI and NotI sites. To reduce the extent of cell death caused by MLKL overexpression toxicity, the full length CMV promoter of pLEX was reduced resulting in minimal CMV promoter driving expression of MLKL. 293T cells were transfected with lipofectamine 2000 reagent according to manufacturer suggestions. Briefly, each transfection required a 90% confluent 10 cm plate of 293T cells in 14 ml DMEM media (10% FBS, 2% PS). Cells were transfected with 2 μg plasmid (MLKL or empty vector), 1.5 μg pSPAX1, 0.5 μg MD2.G with 12 μl transfection reagent in 200 μl Opti-Mem. Virus was collected after 48 h 37 °C incubation and sterile filtered with 0.22 μm syringe filter to remove cell debris. Each 10 cm plate of 293T cells produced enough virus to transduce 3–4 plates of BMDMs. Mouse bone marrow was plated at 2–3 million undifferentiated cells per 10 cm non-tissue culture treated petri dish with 14 ml BMDM media and virus was added during initial plating of bone marrow. Cells would normally be selected with puromycin at 5 μg/ml for 48 h starting on day 5; however, we found that puromycin selection affected cell death sensitivity and thus cells were not puromycin selected for MLKL overexpression experiments. Cells were counted, re-plated in complete RPMI on day 7 of differentiation and rested overnight before experiments.

### Next generation sequencing

Total RNA was isolated from resting BMDMs using TRIzol and used to make a directional cDNA library using TrueSeq kit. Seventy-five bp pair-end reads from cDNA libraries were generated on MiSeq (Illumina) and aligned using TopHat2 and Cufflinks software. Log-transformed values were displayed by heat map. The data are available at the National Center for Biotechnology Information Gene Expression Omnibus:

https://www.ncbi.nlm.nih.gov/geo/query/acc.cgi?acc = GSE83885

### Primers

Primers for mouse IRF7: (F) 5′′-CTTCAGCACTTTCTTCCGAGA-3′, (R) 5′-TGTAGTGTGGTGACCCTTGC-3′; ISG15: (F) 5′-GAGCTAGAGCCTGCAGCAAT-3′, (R) 5′-TTCTGGGCAATCTGCTTCTT-3′, MX1: (F) 5′- TCTGAGGAGAGCCAGACGAT-3′, (R) 5′-ACTCTGGTCCCCAATGACAG-3′, GAPDH: (F) 5′- GGAGAGTGTTTCCTCGTCCC-3′, (R) 5′-TTCCCATTCTCGGCCTTGAC- 3′. TICAM1 (F) 5′- TACCAGCTAAGACCCCTACA-3′, (R) 5′-GTCCCTTTCCAAGGCACCTA- 3′, primers for human IRF7: (F) 5′-CTTGGCTCCTGAGAGGGCAG-3′, (R) 5′-CGAAGTGCTTCCAGGGCA-3′; human ISG15: (F) 5′-TCCTGCTGGTGGTGGACAA-3′, (R) 5′-TTGTTATTCCTCACCAGGATGCT-3′; human MX1: (F) 5′-GTGCATTGCAGAAGGTCAGA-3′, (R) 5′-TCAGGAGCCAGCTTAGGTGT-3′; human GAPDH: (F) 5′-GCTCCTCCTGTTCGACAGTCA-3′, (R) 5′-ACCTTCCCCATGGTGTCTGA-3′.

### ELISA

Cell free supernatants were analyzed for IFNβ as published in ref. [51] and TNFα using the DuoSet elisa kit from R&D (DY410).

### Statistical analysis

Values presented as mean ± standard deviation (SD). Error bars in kinetic viability experiments represent the SD from the mean of triplicate samples. Error bars in qPCR experiments represent the SD from duplicate samples. Data for kinetic viability experiments and qPCR are representative of three or more independent experiments. Time point quantifications of cytotoxicity represent SD from three independent experiments and statistical significance was determined using Student two tailed *t*-test: ns is non-significant (*p* > 0.05); *p < 0.05; **p < 0.01; ***p < 0.001. Comparisons are between two conditions indicated by the ends of the solid lines.

## Electronic supplementary material

Supplementary figures
