# Supplementary material for: Constitutive interferon signaling maintains critical threshold of MLKL expression to license necroptosis
Source: Cell Death Differ. 2018 May 21;26(2):332–47. doi: 10.1038/s41418-018-0122-7 (PMC6329789; doi:10.1038/s41418-018-0122-7)
Supplement: Supplementary file 1 — Supplementary figures [file 41418_2018_122_MOESM1_ESM.pdf]

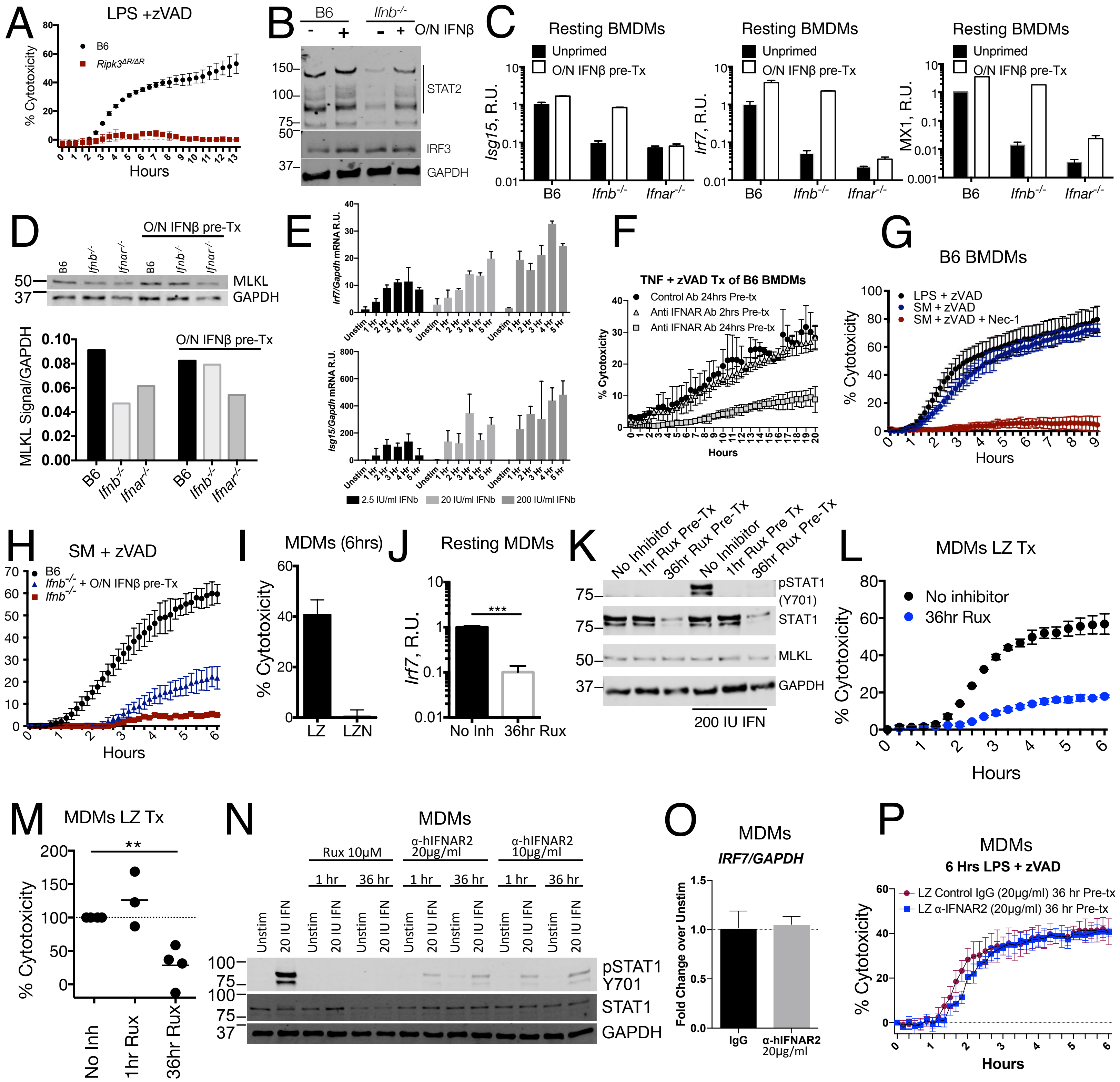

Figure S1. Related to Figure 1

(A) Propidium iodide incorporation as a readout of cytotoxicity, measured every 30 minutes following LPS/zVAD treatment of B6 and *Ripk3*$^{\Delta R/\Delta R}$ BMDMs . (B) Western blot of STAT2, IRF3 and GAPDH from resting B6 and *Ifnb*$^{-/-}$ BMDMs with or without overnight interferon treatment (2.5 IU IFNβ/ml). (C) Quantitative PCR *Isg15*, *Irf7* and *Mx1* mRNA relative to *Gapdh* from resting B6, *Ifnb*$^{-/-}$ and *Ifnar*$^{-/-}$ BMDMs with or without overnight interferon treatment of 2.5 IU/ml IFNβ. (D) Western blot and quantification of MLKL and GAPDH from resting BMDMs of indicated genotype with or without overnight 2.5 IU/ml IFNβ treatment. (E) Quantitative PCR *Isg15*and *Irf7* relative to *Gapdh* from B6 BMDMs treated with indicated doses of recombinant IFNβ for 1-5 hours. (F) Propidium iodide incorporation of B6 BMDMs treated with TNF (50 ng/ml) /zVAD (50 µM) with indicated pre-treatments of IFNAR blocking antibody. (G) Propidium iodide incorporation of B6 BMDMs with LPS/zVAD, SMAC mimetic (SM-164, 1uM) /zVAD or SMAC mimetic (SM-164, 1uM) /zVAD and Nec-1. (H) Propidium iodide incorporation over the first 6 hours of SMAC mimetic (SM-164, 1uM) /zVAD treatment of B6 and *Ifnb*$^{-/-}$ BMDMs that were either untreated or IFN primed (2.5 IU IFNβ/ml) overnight. (I) Propidium iodide incorporation as a measure of cytotoxicity 6 hours following LPS /zVAD (Nec-1 addition indicated) treatment of Human monocyte derived macrophages (MDMs). (J) Quantitative PCR of *Irf7* mRNA relative to *Gapdh* from resting human monocyte derived macrophages (MDMs) that were either untreated or pre-treated for 36 hours with Jak1/2 inhibitor, Ruxolitinib (10uM). (K) Western blot for indicated proteins from MDMs either untreated or pre-treated for one hour or 36 hours with Ruxolitinib (10uM) and then stimulated with 200 IU/ml recombinant human IFNβ for 30 minutes. (L) MDMs were either untreated or pre-treated for 36 hours with Jak1/2 inhibitor, Ruxolitinib (10uM) and then stimulated with LPS/zVAD (LZ) and monitored for viability over six hours. (M) Percent cytotoxicity relative to no inhibitor for MDMs that were either untreated (no inh) or pre-treated for one hour or 36 hours with Ruxolitinib from 3-4 independent experiments. (N) MDMs were pre-treated with either Ruxolitinib (10uM) or human IFNAR blocking antibody (10 or 20 ug/ml) for 1 or 36 hours and stimulated with 20 IU/ml recombinant human IFNβ for 1 hour. (O,P) MDMs were treated with human IFNAR blocking antibody (20 ug/ml) or control IgG (20 ug/ml) for 36 hours and analyzed for resting IRF7 mRNA relative to GAPDH (O) or LPS/zVAD induced cytotoxicity (P). Time point quantifications of cytotoxicity represent SD from three independent experiments and statistical significance was determined using Student two tailed t test: ns is non-significant ($p > 0.05$); *$p < 0.05$; **$p<0.01$; ***$p < 0.001$. Comparisons are between two conditions indicated by the ends of the solid lines. qPCR and all kinetic cytotoxicity assay data are representative of three or more independent experiments. All LPS/zVAD treatments were: LPS [10ng/ml] and zVAD [50uM/ml].

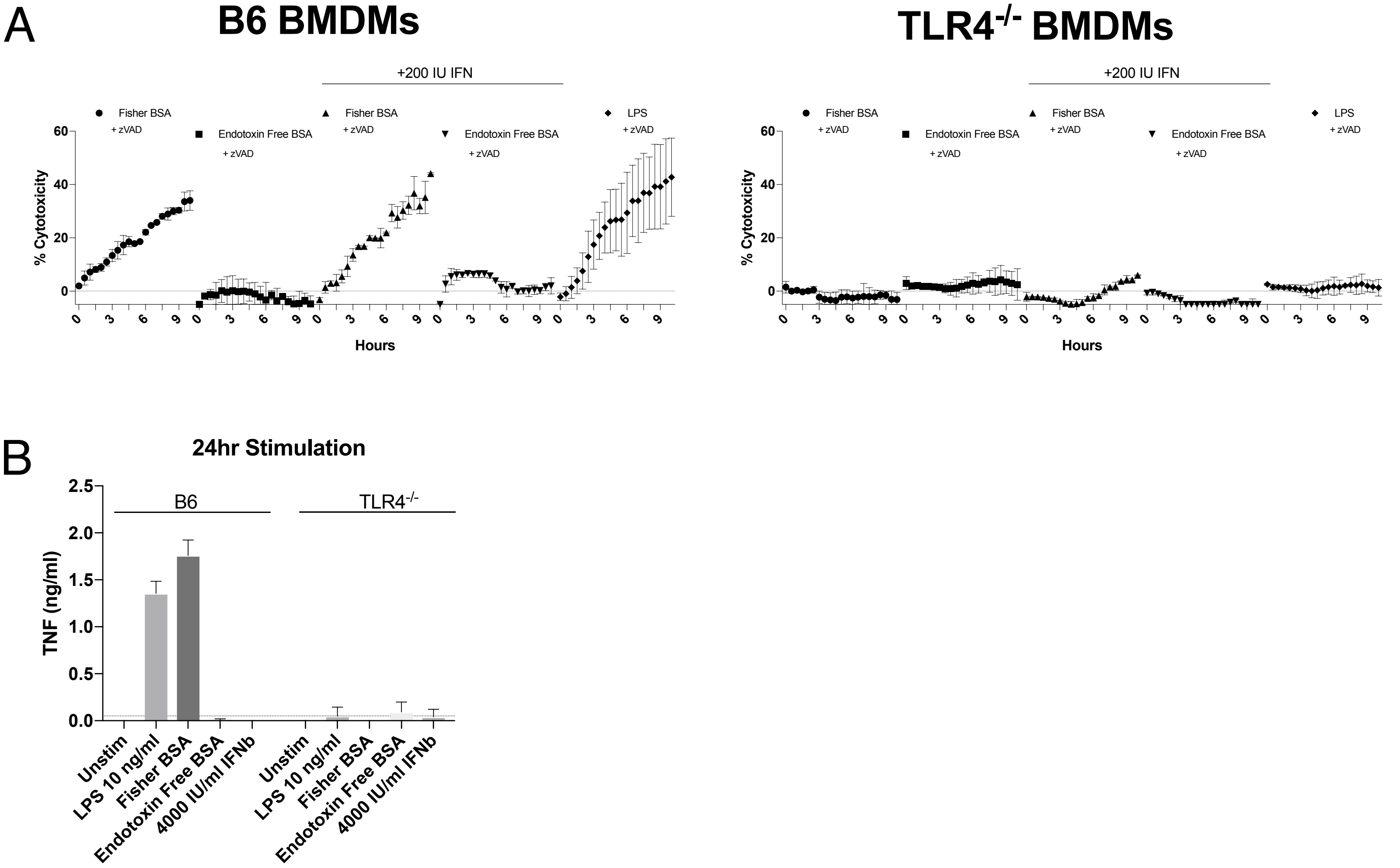

Figure S2. Related to Figure 2

(A) Propidium iodide incorporation as a readout of cytotoxicity, measured every 30 minutes for 10 hours of B6 and *TLR4*$^{-/-}$ BMDMs with indicated treatment conditions. (B) TNF ELISA from supernatant of B6 and *TLR4*$^{-/-}$ BMDMs stimulated 24 hours with indicated conditions (A,B) Concentrations of reagents used in these experiments: LPS (10 ng/ml), Fisher BSA (BP 1600-100) (0.2 ug/ml), endotoxin free BSA from Akron Biotech (AK 8917) (0.2 ug/ml), IFNβ (4000 IU/ml) and zVAD (50uM).

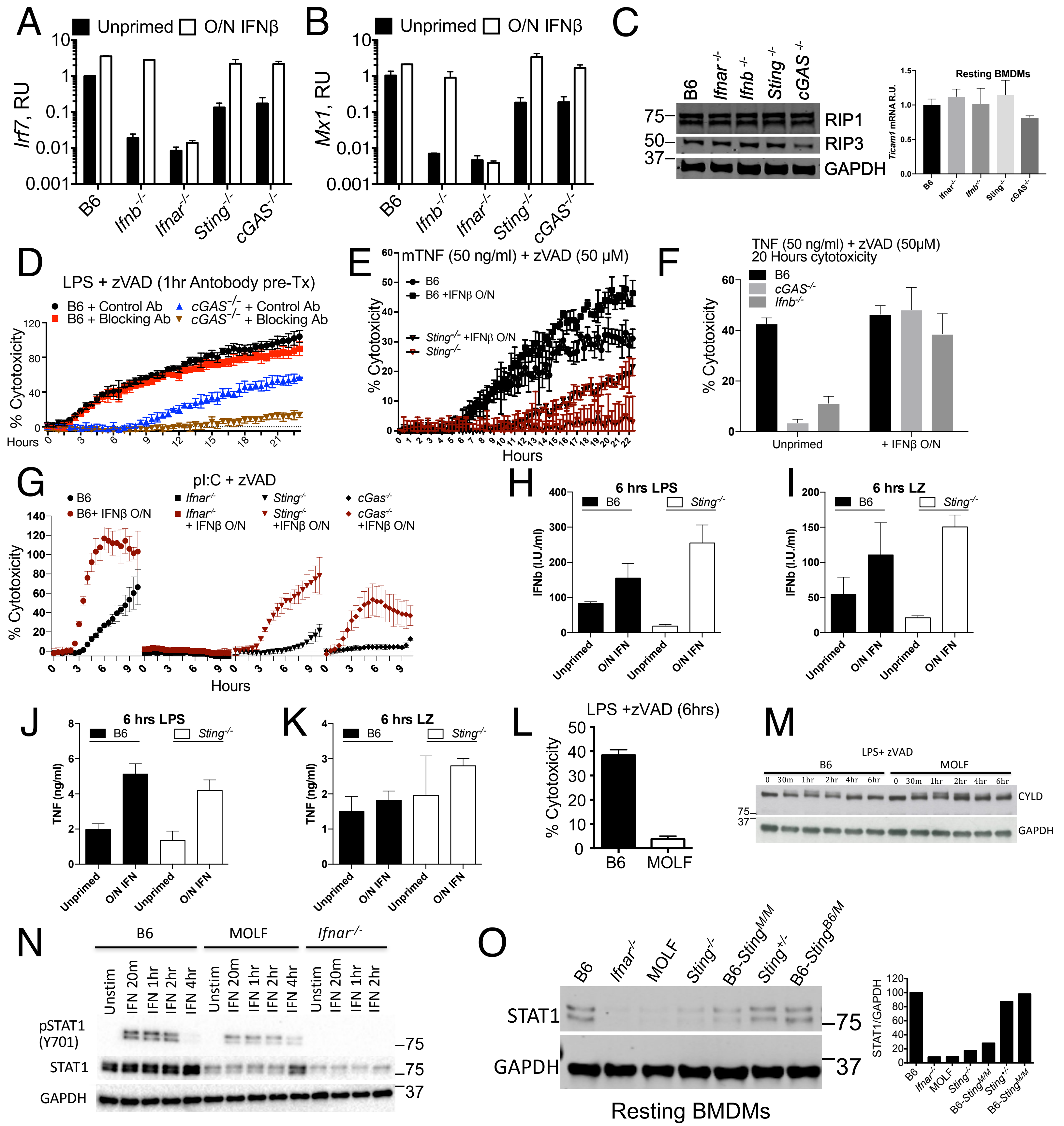

Figure S3. Related to Figure 3

(A,B) Quantitative PCR of *Irf7* (A) and *Mx1* (B) mRNA relative to *Gapdh* from resting B6, *Ifnb*$^{-/-}$, *Ifnar*$^{-/-}$, *Sting*$^{-/-}$, and *cGAS*$^{-/-}$ BMDMs that were untreated or pre-treated with IFNβ overnight (2.5 IU IFNβ/ml). (C)Western blot of resting B6, *Ifnar*$^{-/-}$, *Ifnb*$^{-/-}$, *Sting*$^{-/-}$, and *cGAS*$^{-/-}$ BMDMs for indicated proteins and Quantitative PCR of *Ticam1* relative to *Gapdh* from resting BMDMs. (D) B6 and cGAS$^{-/-}$ BMDMs were pretreated one hour with MAR1 IFNAR blocking or isotype control antibody and monitored for LPS/zVAD induced cytotoxicity. (E) B6 and *Sting*$^{-/-}$ with or without overnight IFNβ treatment (2.5IU/ml) were stimulated with mouse TNF (50 ng/ml) and zVAD (50 uM) and monitored for viability over time. (F) B6, *cGAS*$^{-/-}$ and *Ifnb*$^{-/-}$ BMDMs with or without overnight IFNβ treatment (2.5IU/ml) were stimulated with TNF (50 ng/ml) and zVAD (50 uM) and measured for percentage PI positive cells 20 hours after stimulation. (G) BMDMs of indicated genotype with or without overnight IFNβ treatment (2.5IU/ml) were stimulated with pI:C (25ug/ml) and zVAD (50 uM) and measured for cytotoxicity over time by quantifying the number of PI positive nuclei using an imaging cytometer. (H-K) B6, and Sting$^{-/-}$ BMDMs with or without overnight 2.5 IU IFNβ/ml treatment were stimulated with LPS (H,J) or LPS/zVAD (I, K) for six hours and supernatant IFNβ or TNFα was measured by ELISA. (L) Propidium iodide incorporation as a measure of cytotoxicity 6 hours following LPS /zVAD treatment of B6 and MOLF BMDMs. (M) B6 and MOLF BMDMs were treated with LPS /zVAD for indicated durations and analyzed for CYLD and GAPDH by western blotting. (N) B6, MOLF and *Ifnar*$^{-/-}$ BMDMs were stimulated with 100 IU recombinant IFNβ for indicated time points and analyzed by wester blotting for pSTAT1, STAT1 and GAPDH. (O) Western blot and quantification of STAT1 and GAPDH from unstimulated BMDMs of indicated genotype. All data are representative of three or more independent experiments. All LPS/zVAD treatments were: LPS [10ng/ml] and zVAD [50uM/ml].

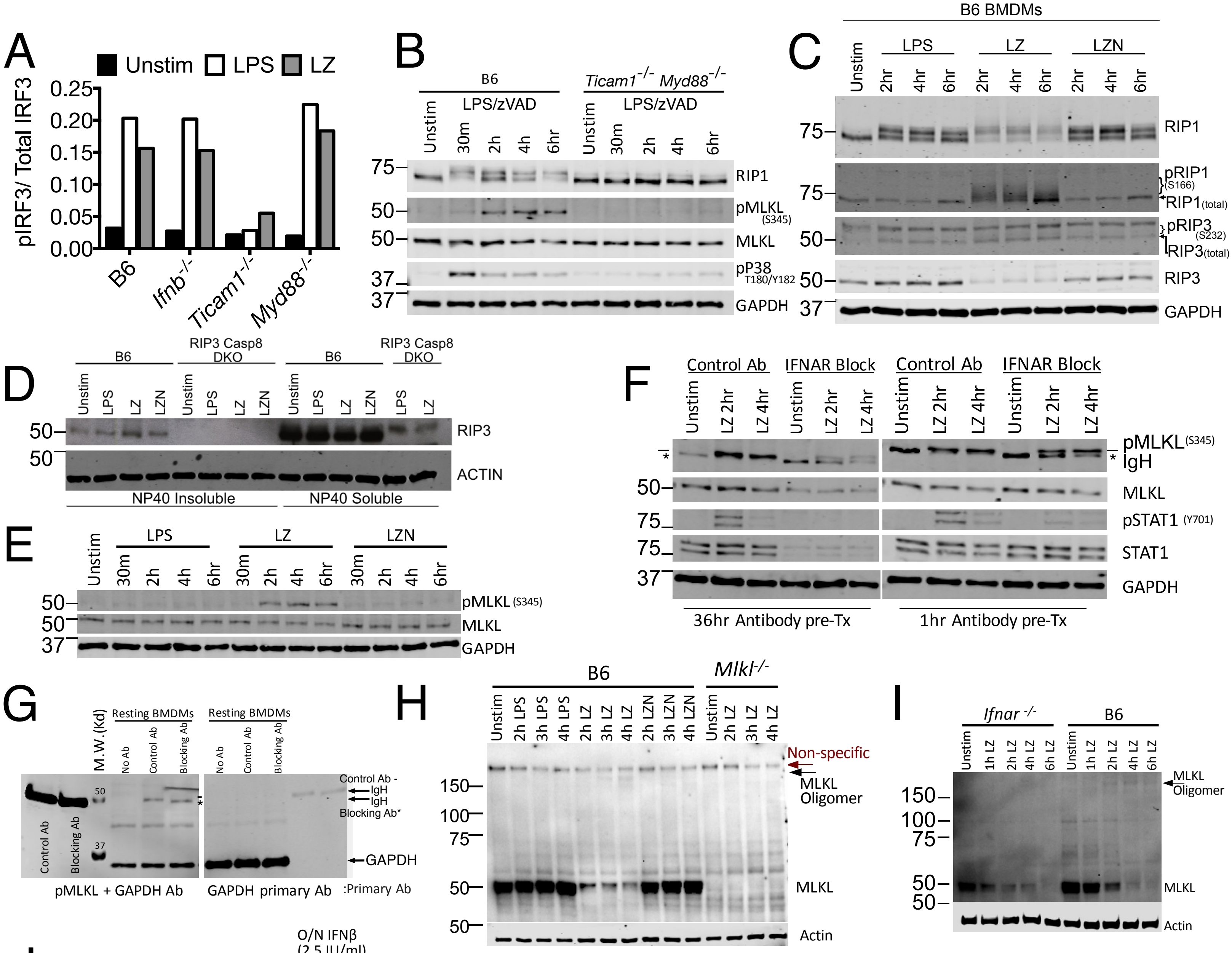

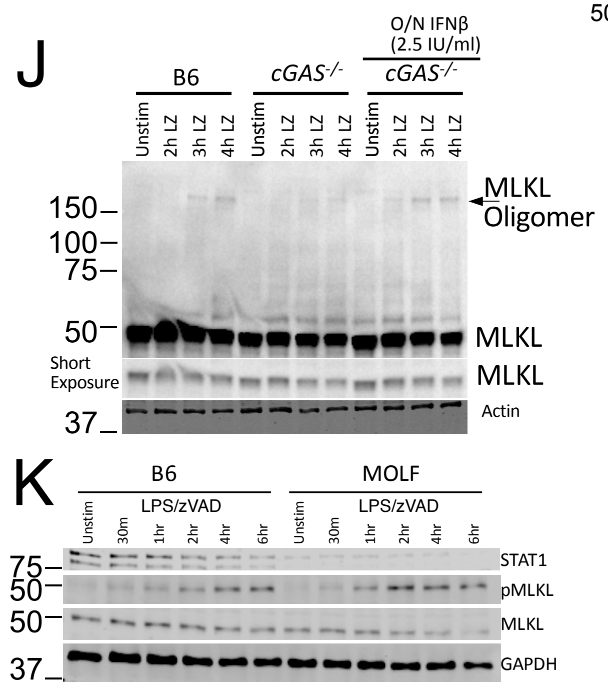

Figure S4. Related to Figure 4
(A) Quantification of pIRF3 signal relative to total IRF3 from Figure 4A (B) Western blots of indicated proteins from B6 and *Ticam1$^{-/-}$/Myd88$^{-/-}$* BMDMs stimulated with LPS/zVAD for various time points. (C) Western blots of indicated proteins from B6 BMDMs that were stimulated with LPS, LPS/zVAD or LPS/zVAD/Nec-1 for various time points. (D) Western blot of RIP3 and Actin from B6 and *RIP3$^{-/-}$/ Casp8$^{-/-}$* BMDMs treated with indicated conditions for 2 hours. This blot is meant to show signal specificity of the RIP3 antibody. Both NP-40 insoluble "necrosome" and NP-40 soluble lysates run on the same gel (E) Western blots of pMLKL, MLKL and GAPDH from B6 BMDMs treated with indicated conditions. (F) Western blots for indicated proteins from B6 BMDMs that were treated with antibody (MAR1 IFNAR blocking or isotype control antibody) 36 hours or only one hour prior to LPS /zVAD treatment. Heavy chain from blocking or control antibody is labeled by IgH and appears below the pMLKL specific band. (G) Western blot of resting B6 BMDMs untreated or treated with either control or IFNAR blocking antibody for one hour. The same quantity of recombinant antibody (blocking or control) added to the cells was loaded directly into the gel on the left and right hand sides of the gel. The membrane was incubated with either pMLKL antibody or GAPDH as indicated and then developed with the same anti rabbit 800nm antibody. The signal shows that the pMLKL antibody strongly reacts with the heavy chain of the IFNAR blocking and control antibody, explaining the nonspecific band appearing below the pMLKL signal in (F). (H) Non reducing western blot for MLKL and ACTIN in B6 and *Mlkl$^{-/-}$* BMDMs treated with indicated conditions. (I) Non reducing western blot for MLKL and ACTIN in *Ifnar$^{-/-}$* and B6 BMDMs treated with LPS/zVAD for indicated time-points. (J) Non-reducing western blot featuring MLKL oligomers from B6 and *cGAS$^{-/-}$* BMDMs (with or without overnight 2.5 IU/ml IFNβ) treated with LPS /zVAD for indicated times. (K) Western blots of indicated proteins from B6 and MOLF BMDMs stimulated with LPS/zVAD for indicated time points. All data are representative of three or more independent experiments. All LPS/zVAD treatments were: LPS [10ng/ml] and zVAD [50uM/ml].

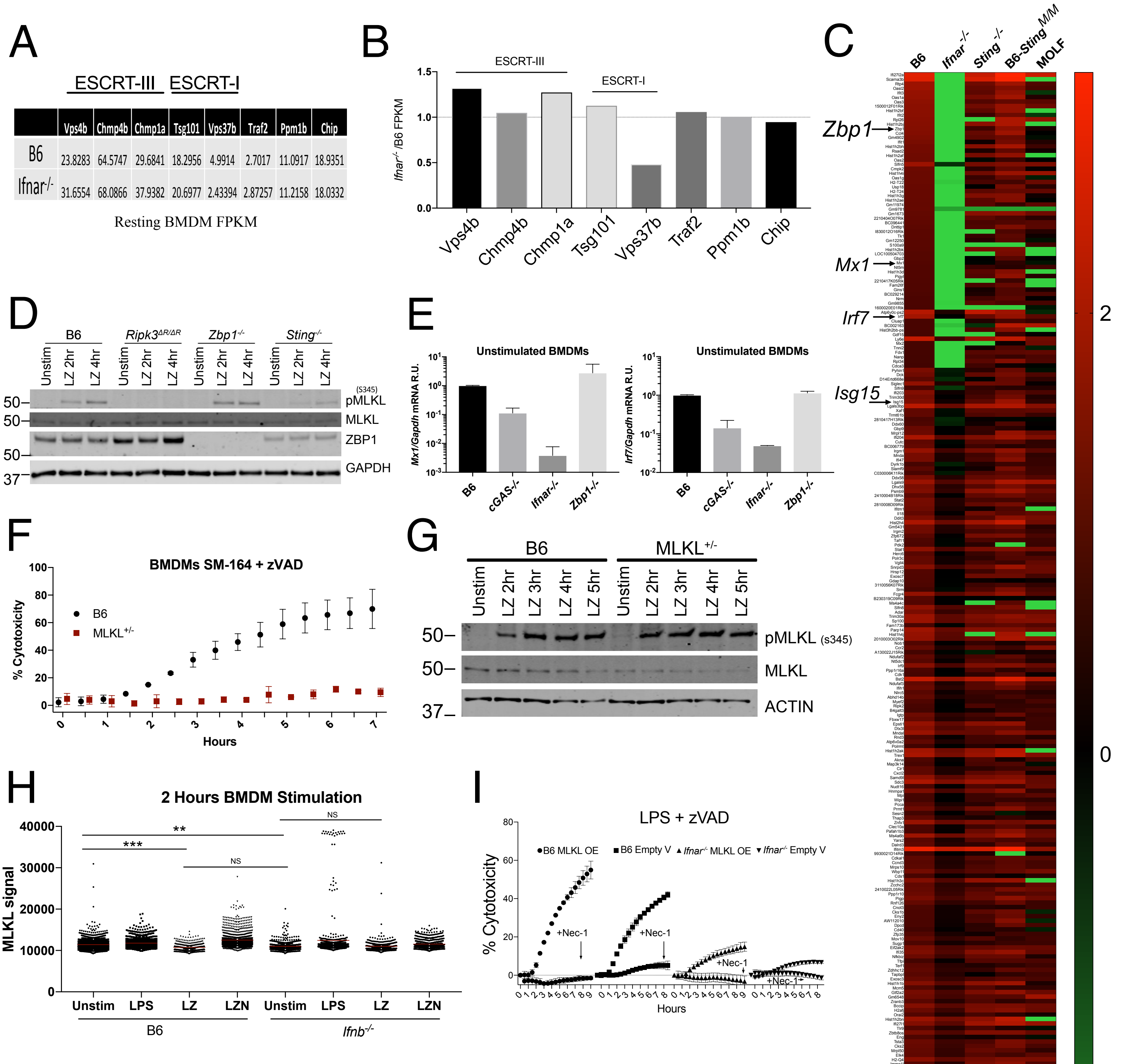

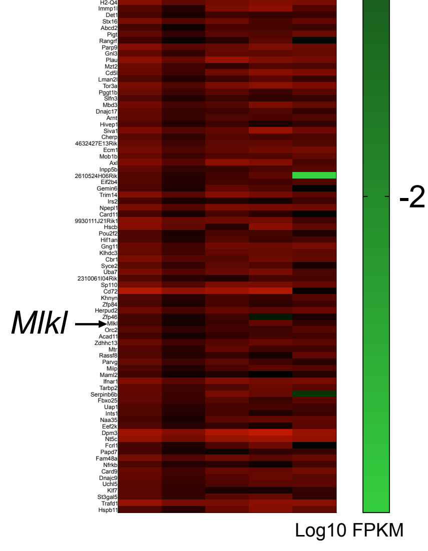

Figure S5. Related to Figure 5

(A,B) Resting state expression of various known inhibitors of necroptosis in B6 and *Ifnar*$^{-/-}$ BMDMs. (A) FPKM values from RNA sequencing of *Ifnar*$^{-/-}$ and B6 BMDMs and (B) ratio of FPKM values from RNA sequencing of *Ifnar*$^{-/-}$ BMDMs / B6 BMDMs where values greater than one indicate higher expression in *Ifnar*$^{-/-}$ compared to B6. (C) RNA sequencing of resting BMDMs showing top 300 genes (>4 FPKM in B6) down-regulated in *Ifnar*$^{-/-}$ BMDMs compared to B6. Resting gene expression is also included for *Sting*$^{-/-}$, and *Sting*$^{MOLF/MOLF}$ and MOLF BMDMs. Certain genes of interest (*Zbp1*, *Mx1*, *Irf7*, *Isg15* and *Mlkl*) are pointed out with arrows. (D) Western blots of pMLKL, MLKL, ZBP1 and GAPDH of BMDMs from indicated genotypes stimulated with LPS/zVAD. (E) Resting BMDM qPCR from B6, *cGAS*$^{-/-}$, *Ifnar*$^{-/-}$ and *Zbp1*$^{-/-}$ BMDMs. (F) 7 hrs time-course of viability of SM-164 (1uM) /zVAD (50uM) treated B6 and Mlkl$^{+/-}$ BMDMs. (G) Western blot of indicated pMLKL, MLKL and ACTIN of B6 and *Mlkl*$^{+/-}$ BMDMs stimulated with LPS/zVAD for various time-points. (H) High content imaging of endogenous MLKL in PFA fixed B6 and *Ifnb*$^{-/-}$ BMDMs treated two hours with LPS, LPS/zVAD, LPS/zVAD/Nec-1. (I) B6 and Ifnar$^{-/-}$ BMDMs transduced with either empty virus of MLKL over expressing virus and analyzed for LPS/zVAD and LPS/zVAD/Nec-1 induced cytotoxicity. All LPS/zVAD treatments were: LPS [10ng/ml] and zVAD [50uM/ml]. MLKL signal compared using student two tailed t test: ns is non-significant ($p > 0.05$); $^{*}p < 0.05$; $^{**}p<0.01$; $^{***}p < 0.001$. All western blot and kinetic cytotoxicity data are representative of three or more experiments.
